# Case report: Periorbital pilomatricoma: a rare benign skin tumor misdiagnosed as cellulitis

**DOI:** 10.3389/fopht.2025.1503693

**Published:** 2025-01-23

**Authors:** Rahul Kumar, Jane Z. Spadaro, Alon Kahana

**Affiliations:** ^1^ College of Medicine, Northeast Ohio Medical University, Rootstown, OH, United States; ^2^ Kahana Oculoplastic and Orbital Surgery, Livonia, MI, United States; ^3^ Beaumont Eye Institute, William Beaumont Hospital, Royal Oak, MI, United States; ^4^ Department of Ophthalmology, Oakland University William Beaumont School of Medicine, Rochester, MI, United States

**Keywords:** orbital tumor, preseptal cellulites, folliculomas, hair follicle tumor, brow and lid reconstruction

## Abstract

**Purpose:**

We describe an unusual case of a rapidly progressive pilomatricoma along the left brow, which was initially misdiagnosed and treated as preseptal cellulitis. Although rare, pilomatricomas and other adnexal tumors should be considered in the differential diagnosis of a growing mass near the brow.

**Case presentation:**

A 29-year-old male presented to the emergency department with a progressively enlarging left brow lesion, initially noted 3 weeks prior. Exam revealed an erythematous left subbrow mass that measured 2.5 x 2 cm, with resultant mechanical ptosis. The lesion was initially misdiagnosed and treated as preseptal cellulitis, with concern for abscess. The patient ultimately underwent excisional biopsy of the lesion and pathology revealed pilomatricoma.

**Conclusions:**

Pilomatricoma has similarities to more common skin lesions. Lack of pain or tenderness are important clues against an infectious or inflammatory etiology. Complete surgical excision is therapeutic and allows for diagnostic confirmation. Histopathology is required to rule out pilomatrix carcinoma, a malignant variant.

## 1 Introduction

Pilomatricomas (PMC), also known as pilomatrixomas or calcifying epitheliomas, were first described in 1880 by Malherbe and Chenantais, and further characterized and identified as originating from hair follicle matrix cells in 1961 by Forbis and Helwig (1). These benign subcutaneous tumors, which are more common in children, usually occur on the head, neck, or upper limbs, and can range in diameter from 0.4-3.5 cm (2). PMCs are rare: among benign skin tumors, PMCs have an incidence of 1% (1). As a result, PMCs are commonly misdiagnosed as sebaceous cysts, dermoid cysts, chalazia, and cellulitis/abscess. Pilomatrix carcinoma, the malignant variant of PMC, also arises from proliferating matrix cells as a firm, non-tender lesion (3). Unlike PMC, pilomatrix carcinoma usually occurs later in life and has a high rate of local recurrence after surgical excision (3).

Herein, we describe an unusual case of a rapidly enlarging PMC of the left brow initially diagnosed and treated as an infection.

## 2 Case presentation

**Figure 1:**
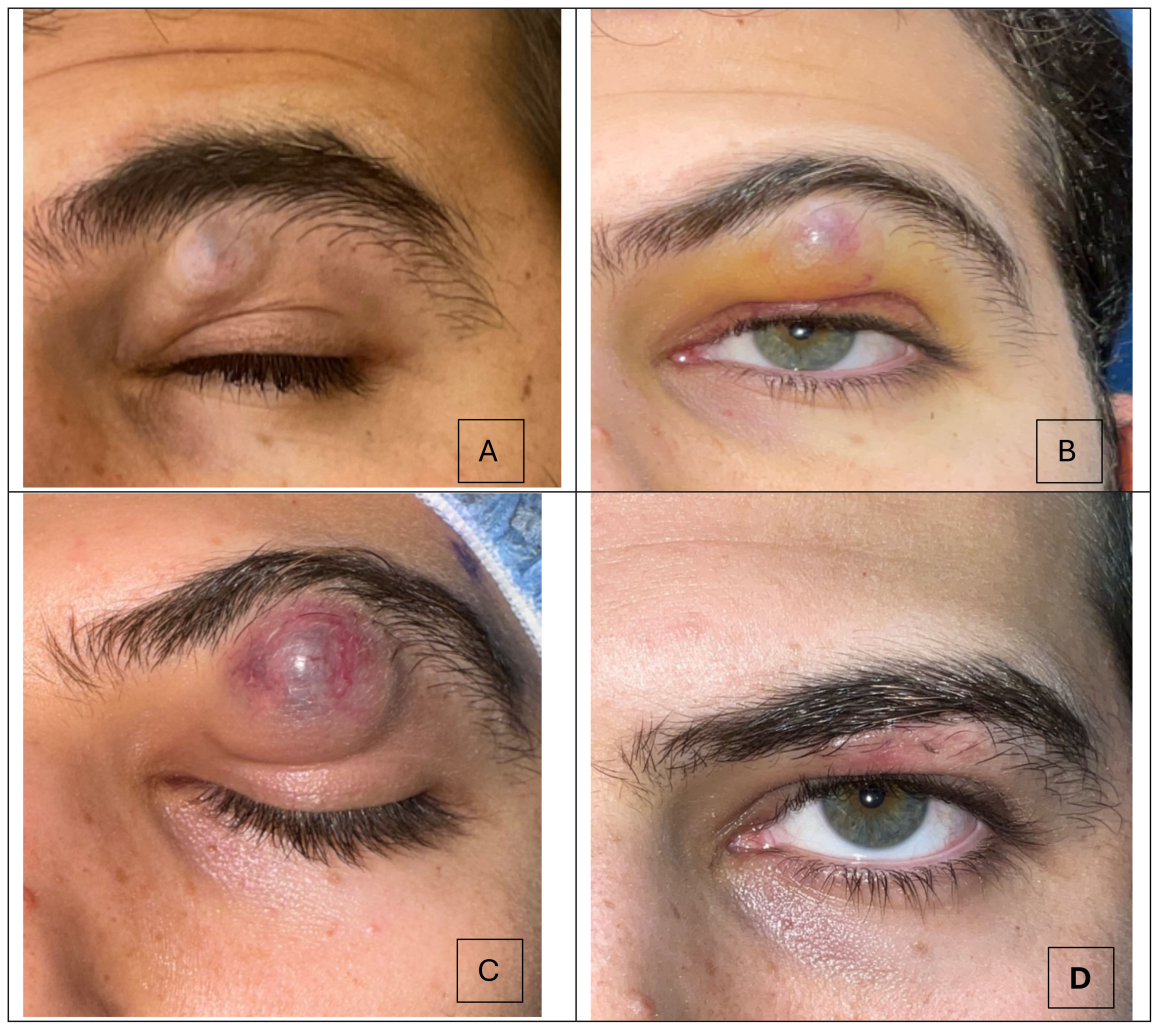
Progression of the subbrow mass over a 30-day timeline. **(A)** Picture taken 3 weeks after the patient first noticed the skin lesion. A subtly discolored mass under the brow is present. **(B)** The lesion continued to progress in size, and measured 2.0 x 1.5 cm **(C)** Preoperative picture where lesion measured 2.5 x 1.0 cm. Ecchymosis and telangiectatic vessels are visible on the lesion. **(D)** Postoperative month 1 demonstrates resolution of the subbrow mass.

A 29-year-old male presented to the clinic with a progressively enlarging left brow mass. He initially presented to his dermatologist, who diagnosed a cystic abscess and attempted to drain the lesion. The lesion continued to increase in size and the patient subsequently presented to the ED, where he was diagnosed with preseptal cellulitis (**Figure 1A**). He was prescribed oral amoxicillin-clavulanate. The patient noted that the lesion continued to grow despite antibiotics, and he was referred for a second opinion.

Upon presentation to the oculoplastic clinic, the patient was noted to have an erythematous 2.0 x 1.5 cm mass, resulting in mechanical ptosis (**Figure 1B**). Otherwise, his visual acuity and intraocular pressures were normal. He had no evidence of proptosis or extraocular dysmotility. External exam revealed a firm, mobile mass without any underlying boney attachments. Importantly, the patient reported no pain, and exam revealed no tenderness. The patient was otherwise in good health and without history of trauma to the area.

**Figure 2:**
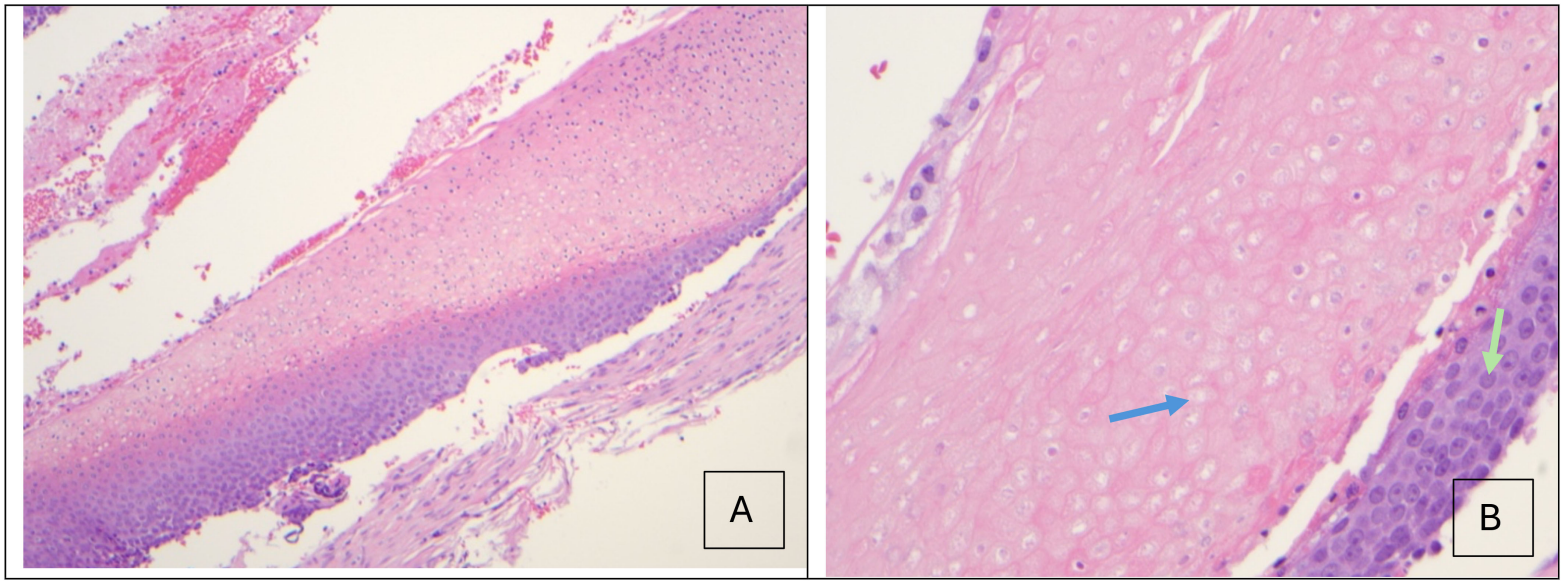
**(A)** Hematoxylin and Eosin (H&E) stain (x10) showing blue basaloid cells, eosinophilic ghost cells, and transition cells with pyknotic nuclei in between. **(B)** Islands of eosinophilic anucleated ghost cells (blue arrow) and the basaloid cells they arise from (green arrow) can be visualized (H&E, x40).

Based on a growing mass in the absence of pain, tenderness or inflammatory signs, a tumor was suspected. He underwent complete excision and biopsy of the lesion, with preservation of skin (**Figure 1C**). Histology revealed islands of eosinophilic ghost cells consistent with pilomatricoma (**Figure 2**). At postoperative month 1, the patient was doing well (**Figure 1D**).

## 3 Discussion and conclusions

Pilomatricomas (PMCs) seen near the brow and eyelid are commonly misdiagnosed as sebaceous cysts, dermoid cysts, chalazia, calcinosis cutis, cellulitis, and basal cell carcinoma due to shared physical features (4). Defining characteristics to differentiate lesions include size and shape of lesion, color, presence and color of discharge, firmness, and tenderness. The lack of pain or tenderness in an otherwise healthy immunocompetent patient selects against an infectious or inflammatory etiology and is more consistent with a tumor.

PMCs can be described as a mass with intact skin overlying ecchymosis and telangiectatic vessels (5). PMCs are firm due to the cellularity of the mass and calcification of ghost/shadow cells. They are usually non-tender and grow slowly. PMCs are anchored to the epidermis but slide freely over the area beneath it (6). The calcified part of the lesion has an angular shape, called the “tent sign”, which can be seen when the overlying skin is stretched to be taut (7). While PMCs tend to be slow-growing, in our case the mass grew rapidly and contained abundant telangiectasis, raising the possibility of a malignant process, e.g. pilomatrix carcinoma, requiring complete excision (8). Given the benign histology, we believe that the rapid grow of this patient’s PMC was due to an intra-lesional hemorrhage.

Histologic analysis of PMCs reveals basophilic basaloid cells giving rise to transition cells with pyknotic nuclei and eosinophilic shadow/ghost cells, with calcifications and both giant cell and mononuclear inflammatory reactions (9, 10).

Pilomatricoma formation is usually caused by somatic activating mutations in the Wnt signaling pathway, specifically in the CTNNB1 gene encoding for β-catenin, a protein found in all active hair follicle matrix cells (11, 12). The mutated β-catenin protein cannot be phosphorylated by WNT-ligand, thus preventing its degradation through the ubiquitin pathway, resulting in downstream expression of proto-oncogens. The combination of mutant β-catenin and additional mutations in proto-oncogenes and/or tumor suppressor genes underlies malignant transformation to pilomatrix carcinoma.

Whenever possible, complete surgical excision is the preferred treatment to minimize risk of recurrence, which is around 2-2.6% (4, 11, 13). The most likely cause of recurrence is incomplete excision.

Pilomatricomas are challenging to diagnose clinically due to their similarities with other common skin lesions. As a result, histological analysis is essential to confirm diagnosis, rule out pilomatrix carcinoma, and reduce the risk of malignant transformation. Although uncommon, pilomatricomas should be considered in the differential diagnosis of growing subcutaneous masses in the periocular region. A key sign is the lack of pain or tenderness on exam.

## Data Availability

The original contributions presented in the study are included in the article/supplementary material. Further inquiries can be directed to the corresponding author.
